# Supplementary material for: Causal Relationship between Aging and Anorexia Nervosa: A White-Matter-Microstructure-Mediated Mendelian Randomization Analysis
Source: Biomedicines. 2024 Aug 16;12(8):1874. doi: 10.3390/biomedicines12081874 (PMC11351342; doi:10.3390/biomedicines12081874)
Supplement: Supplementary file 1 [file biomedicines-12-01874-s001.zip › Supplementary Figure/Figure S4/Singe SNP plot of MR analysis for TL to ubm-b-2070.pdf]

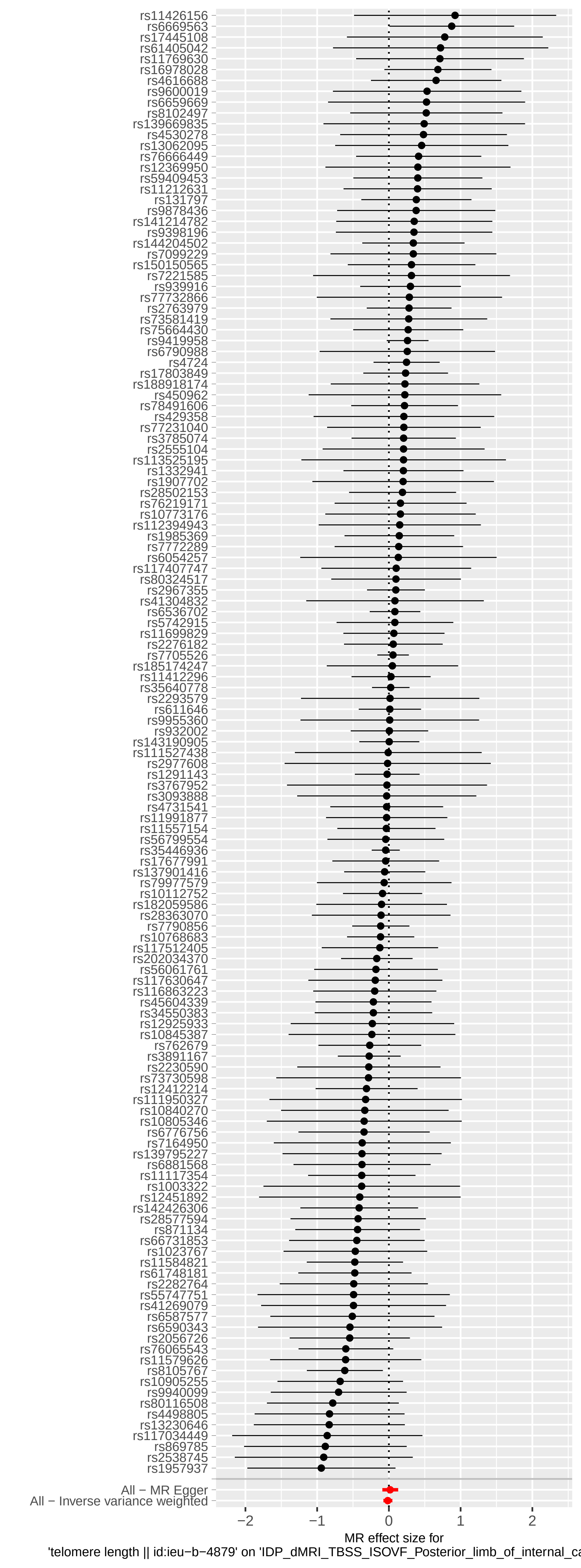
rs11426156
rs6669563
rs17445108
rs61405042
rs11769630
rs16978028
rs4616688
rs9600019
rs6659669
rs8102497
rs139669835
rs4530278
rs13062095
rs76666449
rs12369950
rs59409453
rs11212631
rs131797
rs9878436
rs141214782
rs9398196
rs144204502
rs7099229
rs150150565
rs7221585
rs939916
rs77732866
rs2763979
rs73581419
rs75664430
rs9419958
rs6790988
rs4724
rs17803849
rs188918174
rs450962
rs78491606
rs429358
rs77231040
rs3785074
rs2555104
rs113525195
rs1332941
rs1907702
rs28502153
rs76219171
rs10773176
rs112394943
rs1985369
rs7772289
rs6054257
rs117407747
rs80324517
rs2967355
rs41304832
rs6536702
rs5742915
rs11699829
rs2276182
rs7705526
rs185174247
rs11412296
rs35640778
rs2293579
rs611646
rs9955360
rs932002
rs143190905
rs111527438
rs2977608
rs1291143
rs3767952
rs3093888
rs4731541
rs11991877
rs11557154
rs56799554
rs35446936
rs17677991
rs137901416
rs79977579
rs10112752
rs182059586
rs28363070
rs7790856
rs10768683
rs117512405
rs202034370
rs56061761
rs117630647
rs116863223
rs45604339
rs34550383
rs12925933
rs10845387
rs762679
rs3891167
rs2230590
rs73730598
rs12412214
rs111950327
rs10840270
rs10805346
rs6776756
rs7164950
rs139795227
rs6881568
rs11117354
rs1003322
rs12451892
rs142426306
rs28577594
rs871134
rs66731853
rs1023767
rs11584821
rs61748181
rs2282764
rs55747751
rs41269079
rs6587577
rs6590343
rs2056726
rs76065543
rs11579626
rs8105767
rs10905255
rs9940099
rs80116508
rs4498805
rs13230646
rs117034449
rs869785
rs2538745
rs1957937
All − MR Egger
All − Inverse variance weighted
−2
−1
0
1
2
MR effect size for
'telomere length || id:ieu−b−4879' on 'IDP_dMRI_TBSS_ISOVF_Posterior_limb_of_internal_ca
